# Practice patterns of kidney stone management across European and non-European centers: an in-depth investigation from the European Renal Stone Network (ERSN)

**DOI:** 10.1007/s40620-020-00854-6

**Published:** 2020-09-12

**Authors:** Pietro Manuel Ferraro, Robert Unwin, Olivier Bonny, Giovanni Gambaro

**Affiliations:** 1 U.O.C. Nefrologia, Fondazione Policlinico Universitario A. Gemelli IRCCS, Largo A. Gemelli 8, 00168 Rome, Italy; 2 Università Cattolica del Sacro Cuore, Rome, Italy; 3 Department of Renal Medicine, University College London Medical School, London, UK; 4 Service of Nephrology, Lausanne University Hospital, Lausanne, Switzerland; 5 Division of Nephrology and Dialysis, Department of Medicine, University of Verona, Verona, Italy

**Keywords:** Biomarkers, Bone mineral density, Epidemiology, Guidelines, Mineral metabolism, Nephrolithiasis

## Abstract

**Rationale and objective:**

Kidney stones are a common condition in the general population, however, high-quality evidence for its management is scarce. We propose the creation of an international network with the aim of sharing practice patterns and patient data towards an improvement of our knowledge of the disease.

**Study design:**

Cross-sectional survey.

**Setting and participants:**

An online survey was circulated through several scientific societies. Items were grouped into six domains. Each center’s overall score (OS) was also calculated.

**Analytical approach:**

Chi square and Mann–Whitney tests were performed for differences across centers.

**Results:**

The countries that contributed most were Italy (8.6%), Turkey (6.6%), France and Spain (6.1%). Some type of nutritional work-up was implemented in 62% of centers. A DEXA scan was performed by 46% of centers, whereas some kind of acidification test was performed by 25% of centers. Most centers (80%) implemented blood investigations at least at baseline. With regard to 24-h urine exams, 7 out of 16 were performed by at least 50% of centers. Information on stone composition was collected by 58% of centers. The OS was significantly higher among higher-volume centers compared with lower-volume centers (p = 0.002). Significant differences between EU and non-EU centers were found.

**Limitations:**

Cross-sectional design; no validation on information.

**Conclusions:**

Our survey highlights the potential for the creation of a network of centers that could share information in a common database for observational research and for enrollment of patients in interventional trials.

**Electronic supplementary material:**

The online version of this article (10.1007/s40620-020-00854-6) contains supplementary material, which is available to authorized users.

## Introduction

Kidney stones are increasingly common in the general population [1, 2]. Despite their high recurrence rates and costs [3–5], and their association with severe conditions such as end-stage renal disease and cardiovascular disease [6–11], systematic efforts to standardize their evaluation and follow-up have been scarce. Recently, we reported on the preliminary results of a survey aimed at investigating practice patterns of kidney stone management across centers in Europe with a renowned interest in kidney stones [12]. To obtain a better representation of actual practice patterns even among centers with less expertise in the evaluation and treatment of this condition, we extended the survey to a large, relatively unselected number of centers through involvement of key scientific societies including the European Renal Association-European Dialysis and Transplant Association (ERA-EDTA), the EAU Section of Urolithiasis (EULIS), the European Rare Kidney Disease Reference Network (ERKNet), the European Reference Network for Urogenital Diseases (eUROGEN), and the European Society of Pediatric Nephrology (ESPN). The results of the survey are reported in this paper. The overarching aims of this project would be to: (a) characterize areas of heterogeneity in the management of patients with kidney stones that could be potentially resolved through consensus and/or production of high-quality evidence (b) identify a core of centers which could contribute highly detailed, patient-level clinical and laboratory information as well as biological samples in order to effectively establish a paradigm of precision medicine in the field of kidney stones by integrating phenotypical and “omics” data (c) create a platform for the development of future observational and interventional clinical research in the field of kidney stones. Here, we report on the main results of our survey aimed at investigating current practice patterns across European centers. Since we obtained a rather high number of responses also from non-European centers, we report information from those centers as well.

## Materials and methods

In February 2019, an invitation to complete the survey was sent by e-mail to physicians registered in the mailing lists of ERA-EDTA and EULIS. In May 2019, the same link was circulated through ERKNet, eUROGEN and ESPN. Responses were collected until June 30, 2019. The survey was designed and performed using REDCap. Survey responses were exported as a CSV file and summarized using frequencies and percentages for each item. Responses with missing information on country of origin as well as duplicated responses (in terms of surname, name and e-mail address of the respondent) were removed from the analysis. An “overall score” (OS) was constructed by assigning points for key indicators. Simple comparisons between European and non-European centers were performed with the chi-square test for categorical variables and the rank sum test for continuous variables. All analyses were performed using Stata version 15.1 (Statacorp, TX, USA).

## Results

**Fig. 1:**
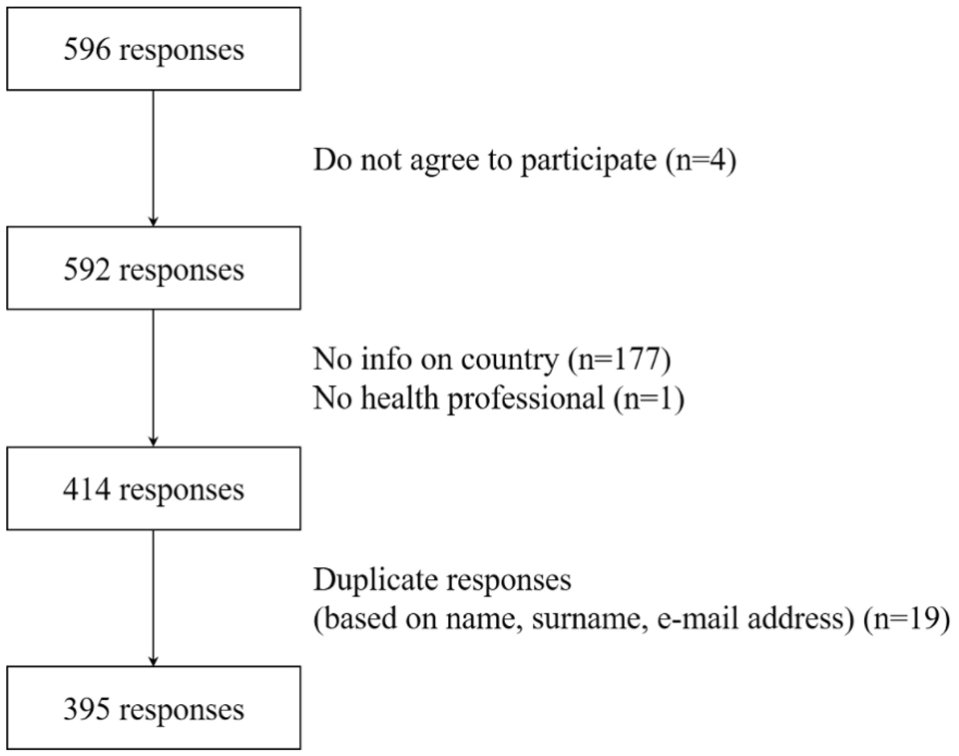
Flowchart of the study

The flowchart of the study is reported in Fig. 1. After exclusions, there were 395 responses available for analysis. Of these, 270 (68%) were from European countries and 125 (32%) from non-European countries.

Results of the “general information” section of the survey are summarized in Table 1 (Supplementary Table 1 for the same information divided by geographic area). The countries that contributed most were Italy (8.6%), Turkey (6.6%), France and Spain (6.1%). The majority (79%) of respondents were adult nephrologists, whereas urologists represented only 6% of respondents. The remaining 15% were mostly pediatric nephrologists. About 50% of respondents saw at least 10 patients per month, although only 10% of centers had very large volumes of patients (more than 30 per month).

**Table 1:** General information

What is your main medical specialty?	
Nephrologist	310 (78.5%)
Urologist	23 (5.8%)
Other	61 (15.4%)
Missing	1 (0.3%)
On average, how many stone patients do you see in your practice?	
Less than 10 per month	195 (49.4%)
Between 10 and 20 per month	117 (29.6%)
Between 20 and 30 per month	31 (7.8%)
More than 30 per month	38 (9.6%)
Missing	14 (3.5%)
How many of those patients represent first visits?	
Less than 10%	125 (31.6%)
Between 10 and 50%	199 (50.4%)
More than 50%	56 (14.2%)
Missing	15 (3.8%)
Are you primarily involved in	
Urological procedures for stone removal	19 (4.8%)
Evaluation and care of unselected patients with stones	209 (52.9%)
Evaluation and care of selected patients with stones (e.g. metabolic stones)	134 (33.9%)
Other	18 (4.6%)
Missing	15 (3.8%)
What is the main source of your stone patients?	
General practitioner	107 (27.1%)
Urologist	117 (29.6%)
General renal outpatient clinic	118 (29.9%)
Other	38 (9.6%)
Missing	15 (3.8%)

Results of the “referral and follow-up criteria” section of the survey are summarized in Table 2 (Supplementary Table 2 for the same information divided by geographic area). Only 15% of centers adopted formal criteria for referral to their stone clinic, but the majority (62%) adopted a formal follow-up scheme which included a visit at 3 months and at 12 months in 39 and 17% of cases, respectively. About 80% of centers performed systematic imaging studies during follow-up, mostly represented by renal ultrasound (73%) and, to a lesser extent, CT scan (22%).

**Table 2:** Referral and follow-up criteria

Do you select the stone patients seen in your clinic based on formal criteria?	
No	314 (79.5%)
Yes	61 (15.4%)
Missing	20 (5.1%)
Do you follow a formal follow-up scheme for your stone patients?	
No	129 (32.7%)
Yes	245 (62.0%)
Missing	21 (5.3%)
Does your follow-up scheme involve visits at 3 months?	
No	241 (61.0%)
Yes	154 (39.0%)
Does your follow-up scheme involve visits at 12 months?	
No	331 (83.8%)
Yes	64 (16.2%)
Does your follow-up scheme involve visits further than 12 months?	
No	387 (98.0%)
Yes	8 (2.0%)
Do you systematically perform imaging studies during follow-up?	
No	59 (14.9%)
Yes	312 (79.0%)
Missing	24 (6.1%)
Which kind of imaging? (choice = Ultrasound)	
No	107 (27.1%)
Yes	288 (72.9%)
Which kind of imaging? (choice = X-ray)	
No	331 (83.8%)
Yes	64 (16.2%)
Which kind of imaging? (choice = Un-enhanced CT scan)	
No	309 (78.2%)
Yes	86 (21.8%)
Which kind of imaging? (choice = Enhanced CT scan)	
No	368 (93.2%)
Yes	27 (6.8%)

Results of the “nutritional inquiry” section of the survey are summarized in Table 3 (Supplementary Table 3 for the same information divided by geographic area). Some type of nutritional work-up was implemented in 62% of centers, with a combination of diet diaries (43%), food-frequency questionnaires/food screeners (20%), and 24-h dietary recalls (17%). A dietitian was employed in clinical practice by 35% of centers. Of note, the nutritional work-up was adopted significantly more frequently among European centers (68 vs 49%, p < 0.001), however the frequency of dietitian employment was similar (38 vs 30%, p = 0.20).

**Table 3:** Nutritional inquiry

Do you perform a nutritional work-up on your stone patients?	
No	122 (30.9%)
Yes	245 (62.0%)
Missing	28 (7.1%)
Which kind of nutritional work-up? (Diet diary)	
No	224 (56.7%)
Yes	171 (43.3%)
Which kind of nutritional work-up? (24 h recall)	
No	331 (83.8%)
Yes	64 (16.2%)
Which kind of nutritional work-up? [Food-frequency questionnaire (full)]	
No	359 (90.9%)
Yes	36 (9.1%)
Which kind of nutritional work-up? [Food-frequency questionnaire (screen)]	
No	354 (89.6%)
Yes	41 (10.4%)
Does your nutritional work-up include evaluation by a dietitian?	
No	227 (57.5%)
Yes	140 (35.4%)
Missing	28 (7.1%)

Results of the “special tests” section of the survey are summarized in Table 4 (Supplementary Table 4 for the same information divided by geographic area). A DEXA scan was performed systematically or based on selected criteria by 46% of centers, the most common criteria for DEXA being hypercalciuria and stone composition. Some kind of acidification test, such as the ammonium chloride and/or furosemide/fludrocortisone test, was performed by 25% of centers. Among European centers, performing a DEXA scan was more common (52 vs 33%, p = 0.003), whereas the use of acidification tests was not significantly different (27 vs 18%, p = 0.12).

**Table 4:** Special tests

Do you perform a DXA scan as part of your metabolic work-up?	
Always	12 (3.0%)
Based on selected criteria	169 (42.8%)
Never	171 (43.3%)
Missing	43 (10.9%)
Which criteria do you follow for DXA scan? (hypercalciuria)	
Unchecked	279 (70.6%)
Checked	116 (29.4%)
Which criteria do you follow for DXA scan? (hypocitraturia)	
Unchecked	363 (91.9%)
Checked	32 (8.1%)
Which criteria do you follow for DXA scan? (stone composition)	
Unchecked	320 (81.0%)
Checked	75 (19.0%)
Do you perform an acidification test as part of your metabolic work-up?	
Always	17 (4.3%)
Based on selected criteria	81 (20.5%)
Never	253 (64.1%)
Missing	44 (11.1%)
Which type of acidification test do you perform? [ammonium chloride (full)]	
Unchecked	366 (92.7%)
Checked	29 (7.3%)
Which type of acidification test do you perform? (ammonium chloride other)	
Unchecked	369 (93.4%)
Checked	26 (6.6%)
Which type of acidification test do you perform? (furosemide/fludrocortisone)	
Unchecked	352 (89.1%)
Checked	43 (10.9%)

Results of the “laboratory investigations” section of the survey are summarized in Table 5 (Supplementary Table 5 for the same information divided by geographic area, Supplementary Tables 6–8 for the list of the individual laboratory exams). Most centers (80%) implemented some blood investigations at least at baseline, whereas spot and 24-h urine investigations were performed in 65 and 72% of cases, respectively. Among the individual laboratory exams, the majority (12 out of 14) of blood parameters surveyed were performed by at least 50% of centers, the only exceptions being 25(OH) vitamin D (2%) and 1,25(OH)_2_ vitamin D (29%). Conversely, for spot urine the only exams performed by at least 50% of centers were pH and sediment examination, whereas urine calcium was performed on spot urine by 47% of centers. With regard to 24-h urine exams, 7 out of 16 were performed by at least 50% of centers, with the most commonly performed exams being calcium (69%), uric acid (63%), oxalate (62%), creatinine (61%) and citrate (59%). At baseline examination, 23% of centers performed two separate 24-h urine collections, with heterogeneous modalities of collection: the majority performed the collection in plain bottles with or without preservatives, whereas 25% used acidified bottles. Only a small percentage (5%) of centers reported the use of software for estimation of urinary supersaturations. Information on stone composition was collected by 58% of centers, 28% with a gold-standard technique (i.e., Fourier-transform infrared analysis and/or X-ray diffraction).

**Table 5:** Laboratory investigations

Does your metabolic work-up include laboratory investigations on blood samples?	
In all patients	278 (70.4%)
In selected patients	37 (9.4%)
No	7 (1.8%)
Missing	73 (18.5%)
Do you perform blood tests:	
Only at baseline	44 (11.1%)
At baseline and follow-up	268 (67.8%)
Only at follow-up	2 (0.5%)
Missing	81 (20.5%)
Does your metabolic work-up include laboratory investigations on spot urine samples?	
In all patients	227 (57.5%)
In selected patients	30 (7.6%)
No	64 (16.2%)
Missing	74 (18.7%)
Do you perform spot urine tests:	
Only at baseline	25 (6.3%)
At baseline and follow-up	222 (56.2%)
Only at follow-up	8 (2.0%)
Missing	140 (35.4%)
Does your metabolic work-up include laboratory investigations on 24 h urine samples?	
In all patients	197 (49.9%)
In selected patients	88 (22.3%)
No	34 (8.6%)
Missing	76 (19.2%)
Do you perform 24 h urine tests:	
Only at baseline	61 (15.4%)
At baseline and follow-up	210 (53.2%)
Only at follow-up	14 (3.5%)
Missing	110 (27.8%)
How many 24 h urine collections per work-up do you perform at baseline?	
One	161 (40.8%)
Two	90 (22.8%)
More than two	18 (4.6%)
Missing	126 (31.9%)
How many 24 h urine collections per work-up do you perform at follow-up?	
One	159 (40.3%)
Two	32 (8.1%)
More than two	33 (8.4%)
Missing	171 (43.3%)
Which kind of 24 h urine collection do you perform? (choice = plain bottle)	
Unchecked	182 (46.1%)
Checked	213 (53.9%)
Which kind of 24 h urine collection do you perform? (choice = acidified bottle)	
Unchecked	299 (75.7%)
Checked	96 (24.3%)
Which kind of 24 h urine collection do you perform? (choice = alkalinized bottle)	
Unchecked	377 (95.4%)
Checked	18 (4.6%)
Which kind of 24 h urine collection do you perform? (choice = bottle with antibacte	
Unchecked	368 (93.2%)
Checked	27 (6.8%)
Do you routinely obtain supersaturation indices from 24 h urine collections?	
No	265 (67.1%)
Yes	19 (4.8%)
Missing	111 (28.1%)
Which supersatiuration indices do you obtain? (choice = activity products)	
Unchecked	384 (97.2%)
Checked	11 (2.8%)
Which supersatiuration indices do you obtain? (choice = EQUIL-2)	
Unchecked	392 (99.2%)
Checked	3 (0.8%)
Which supersatiuration indices do you obtain? (choice = JESS)	
Unchecked	395 (100.0%)
Which supersatiuration indices do you obtain? (choice = PSF)	
Unchecked	394 (99.7%)
Checked	1 (0.3%)
Which supersatiuration indices do you obtain? (choice = Betas)	
Unchecked	395 (100.0%)
Do you routinely obtain stone composition analysis?	
No	99 (25.1%)
Yes	229 (58.0%)
Missing	67 (17.0%)
Which kind of stone composition analysis? (choice = IR-spectroscopy)	
Unchecked	307 (77.7%)
Checked	88 (22.3%)
Which kind of stone composition analysis? (choice = X-ray diffraction)	
Unchecked	360 (91.1%)
Checked	35 (8.9%)
Which kind of stone composition analysis? (choice = chemical analysis)	
Unchecked	247 (62.5%)
Checked	148 (37.5%)

In our survey, we also asked centers what type of information they collected with regard to several domains, including demographics (Supplementary Table 9), general medical status (Supplementary Table 10), kidney stone history and status (Supplementary Tables 11 and 12), diet and lifestyle (Supplementary Table 13), and physical examination (Supplementary Table 14). Information on the availability of administrative and research tools was obtained (Supplementary Table 15). Finally, we asked centers how they would define an incident stone event (Table 6 and Supplementary Table 16).

**Table 6:** Incident stone event

How do you define an incident stone event? (choice = new stone formation)	
Unchecked	121 (30.6%)
Checked	274 (69.4%)
How do you define an incident stone event? (choice = growth of a previous stone)	
Unchecked	256 (64.8%)
Checked	139 (35.2%)
How do you define an incident stone event? (choice = stone expulsion)	
Unchecked	230 (58.2%)
Checked	165 (41.8%)
How do you define an incident stone event? (choice = urological intervention)	
Unchecked	237 (60.0%)
Checked	158 (40.0%)
How do you define an incident stone event? (choice = renal colic)	
Unchecked	195 (49.4%)
Checked	200 (50.6%)

The criteria used for the creation of the OS are reported in Supplementary Table 17. OS appeared to be slightly but significantly higher among European centers (median 8, interquartile range 6–11) compared with non-European centers (median 7, interquartile range 4–9) (p < 0.001). Furthermore, OS was significantly higher among higher-volume centers (e.g., those following more than 10 patients per month, median 8, interquartile range 6–11) compared with lower-volume centers (median 7, interquartile range 5–9) (p = 0.002).

Differences between observed pattern and guideline recommendations are reported in Table 7.

**Table 7:** Differences between guideline recommendations and actual practice patterns

	All centers (%)	European centers (%)	Non-European centers (%)	Centers led by urologists (%)	Centers led by nephrologists (%)
Dietary habits	62	73	54	68	70
Ultrasound	74	73	75	83	71
Serum creatinine	79	84	70	74	79
Serum calcium	78	83	66	65	79
Uric acid	78	82	69	65	79
Dipstick on spot urine	27	29	21	9	27
Urine sediment	50	54	41	51	35
Stone composition	58	75	55	94	66

Based on EAU guidelines [24].

## Discussion

This study expands on our previous work in which we surveyed 24 medical centers from Europe about their current practice patterns in the management of patients with kidney stones [12]. Since the previous survey only included centers with renowned expertise in the metabolic management of such patients, the results could not necessarily reflect actual practices across Europe. With the aim of improving external validity, we enrolled a larger number of centers in a more unbiased way, namely by accessing the mailing list of major European scientific societies in the field of nephrology and urology. When comparing our current results with our previous study, there are a number of interesting findings: first, in the present, much larger sample of centers, the proportion of those reporting seeing less than 10 patients per month increased significantly from 13 to 49%; similarly, the proportion of centers with formal referral criteria decreased from 21 to 15%. Taken together, these findings are indirect proof that the current survey was indeed successful in sampling a less selected group of centers than the original.

In this study, we found that higher-volume centers had higher values of OS compared with lower-volume centers. Although not unexpected, this finding underlines the need for larger centers to take the lead in guiding the process of standardization of clinical and research procedures in the field.

Interestingly, the proportion of participating urological centers decreased dramatically from 21 to 6% despite circulating the survey to two mailing lists of urologists (EULIS and eUROGEN), suggesting that the engagement of urologists in the metabolic evaluation and follow-up of stone patients might be lower in general compared with nephrologists; however, the urological centers that did participate showed similar adherence to guidelines compared with nephrological centers (Table 7). Furthermore, we must take into account that we did not have access to the mailing list of the main European scientific society of Urology (European Association of Urology, EAU). However, EULIS is the urolithiasis section of the EAU, which probably affiliates most of the urologists interested in renal stone treatment. It is worth noting that a relatively large number of surveys was not analyzed due to missing information on country. This is the result of the choice to avoid required fields in order to collect as much information as possible from participating centers; however, since the vast majority of surveys missing information on country was also missing information on most of the survey items, we believe that the final results were not distorted.

Our study confirms that nutritional investigation is commonly performed in stone formers, as it should be given the large impact that dietary habits have on the risk of stone formation [13–15]. Interestingly, we found that the proportion of centers performing nutritional investigation was significantly higher among European centers, however these findings are difficult to interpret given the heterogeneity of health system policies in place across geographical areas.

Another interesting finding is the relatively common use of DEXA scans in stone centers. It is well-established that stone formers are at increased risk of reduced bone density and bone fractures [16–20], mainly due to a status of negative calcium balance [21]. It is thus important to investigate the bone status in such patients, especially those with suspect features such as high urine calcium, low urine citrate and high urine pH. In fact, distal tubular acidosis represents a potential risk factor for both kidney stones and reduced bone density given the positive acid balance that it entails [22]. An acidification test is necessary to diagnose an incomplete form of distal tubular acidosis [23]; unfortunately, only about 1 in 4 centers performed acidification tests, meaning that a number of missed diagnoses is expected for this potentially treatable condition.

It is unfortunate to observe that in a significant number of centers, both within and outside Europe, the practice patterns for the investigation of stone patients deviate from what the EAU guidelines suggest [24]. For instance, guidelines suggest investigating dietary habits in all stone formers, either with known or unknown stone composition, as well as performing ultrasound, blood analysis (serum creatinine, calcium) and a dipstick test on spot urine (Table 7). In particular, a very low number of centers performed urinalysis with microscopy, an inexpensive test that can provide the clinician with useful information such as urine pH, specific gravity and presence of crystalluria or proteinuria. The small number of centers performing a proper stone composition analysis is also worth noting.

It is puzzling that 24 h urine collection protocols are so heterogeneous among centers. This underlines the need for a consensus statement on how (acidified or native urine; preservatives or not) and how many times (number of collections) urine samples should be collected. Of note, changes in the way urine collection is performed have an important preanalytical impact on the results [25].

Finally, based on our investigation, we believe that there is the potential to create a network of centers that could share information in a common database for observational research as well as a platform for enrollment of well-phenotyped patients in interventional trials. In fact, a large proportion of centers used electronic records to store patient information and some also had biobanks in place with valuable biological samples. We believe that such an effort would be critical for the development of precision medicine in the field of kidney stones, with the aim of reducing the burden of recurrence and systemic complications of this condition.

## Electronic supplementary material

Below is the link to the electronic supplementary material.

Supplementary file1 (DOCX 61 kb)

Supplementary file2 (DOCX 24 kb)

## Data Availability

Dtransparency.
