# Supplementary material for: Practice patterns of kidney stone management across European and non-European centers: an in-depth investigation from the European Renal Stone Network (ERSN)
Source: J Nephrol. 2020 Sep 12;34(4):1337–46. doi: 10.1007/s40620-020-00854-6 (PMC8357688; doi:10.1007/s40620-020-00854-6)
Supplement: Supplementary file 1 — Supplementary file1 (DOCX 61 kb) [file 40620_2020_854_MOESM1_ESM.docx]

**Practice patterns of kidney stones management across European and non-European centers: an in-depth investigation from the European Renal Stone Network (ERSN)**

Journal of Nephrology

Pietro Manuel Ferraro, Robert Unwin, Olivier Bonny, Giovanni Gambaro

Corresponding author:

Pietro Manuel Ferraro, MD MSc PhD

U.O.C. Nefrologia, Fondazione Policlinico Universitario A. Gemelli IRCCS

Università Cattolica del Sacro Cuore

Via G. Moscati 31, 00168, Roma, Italia

E-mail: pietromanuel.ferraro@unicatt.it

Tel: +39-06-3015-9981; Fax: +39-06-3015-9423

**Supplementary Table 1. General information divided by geographic area**

|  | **non-EU** | **EU** | **p-value** |
| --- | --- | --- | --- |
|  | 125 | 270 |  |
| **What is your main medical specialty?** |  |  | 0.14 |
| Nephrologist | 102 (81.6%) | 208 (77.0%) |  |
| Urologist | 3 (2.4%) | 20 (7.4%) |  |
| Other | 19 (15.2%) | 42 (15.6%) |  |
| Missing | 1 (0.8%) | 0 (0.0%) |  |
| **On average, how many stone patients do you see in your practice?** |  |  | 0.74 |
| Less than 10 per month | 57 (45.6%) | 138 (51.1%) |  |
| Between 10 and 20 per month | 38 (30.4%) | 79 (29.3%) |  |
| Between 20 and 30 per month | 11 (8.8%) | 20 (7.4%) |  |
| More than 30 per month | 14 (11.2%) | 24 (8.9%) |  |
| Missing | 5 (4.0%) | 9 (3.3%) |  |
| **How many of those patients represent first visits?** |  |  | 0.074 |
| Less than 10% | 37 (29.6%) | 88 (32.6%) |  |
| Between 10 and 50% | 58 (46.4%) | 141 (52.2%) |  |
| More than 50% | 25 (20.0%) | 31 (11.5%) |  |
| Missing | 5 (4.0%) | 10 (3.7%) |  |
| **Are you primarily involved in:** |  |  | 0.079 |
| Urological procedures for stone removal | 2 (1.6%) | 17 (6.3%) |  |
| Evaluation and care of unselected patients with stones | 75 (60.0%) | 134 (49.6%) |  |
| Evaluation and care of selected patients with stones | 39 (31.2%) | 95 (35.2%) |  |
| Other | 4 (3.2%) | 14 (5.2%) |  |
| Missing | 5 (4.0%) | 10 (3.7%) |  |
| **What is the main source of your stone patients?** |  |  | <0.001 |
| General practitioner | 41 (32.8%) | 66 (24.4%) |  |
| Urologist | 20 (16.0%) | 97 (35.9%) |  |
| General renal outpatient clinic | 49 (39.2%) | 69 (25.6%) |  |
| Other | 10 (8.0%) | 28 (10.4%) |  |
| Missing | 5 (4.0%) | 10 (3.7%) |  |

**Supplementary Table 2. Referral and follow-up criteria divided by geographic area**

|  | **non-EU** | **EU** | **p-value** |
| --- | --- | --- | --- |
|  | 125 | 270 |  |
| **Do you select the stone patients seen in your clinic based on formal criteria?** |  |  | 0.062 |
| No | 105 (84.0%) | 209 (77.4%) |  |
| Yes | 13 (10.4%) | 48 (17.8%) |  |
| Missing | 7 (5.6%) | 13 (4.8%) |  |
| **Do you follow a formal follow-up scheme for your stone patients?** |  |  | 0.53 |
| No | 38 (30.4%) | 91 (33.7%) |  |
| Yes | 80 (64.0%) | 165 (61.1%) |  |
| Missing | 7 (5.6%) | 14 (5.2%) |  |
| **Does your follow-up scheme involve visits at 3 months?** |  |  | <0.001 |
| Unchecked | 61 (48.8%) | 180 (66.7%) |  |
| Checked | 64 (51.2%) | 90 (33.3%) |  |
| **Does your follow-up scheme involve visits at 12 months** |  |  | <0.001 |
| Unchecked | 117 (93.6%) | 214 (79.3%) |  |
| Checked | 8 (6.4%) | 56 (20.7%) |  |
| **Does your follow-up scheme involve visits further than 12 months?** |  |  | 0.72 |
| Unchecked | 122 (97.6%) | 265 (98.1%) |  |
| Checked | 3 (2.4%) | 5 (1.9%) |  |
| **Do you systematically perform imaging studies during follow-up?** |  |  | 0.044 |
| No | 12 (9.6%) | 47 (17.4%) |  |
| Yes | 105 (84.0%) | 207 (76.7%) |  |
| Missing | 8 (6.4%) | 16 (5.9%) |  |
| **Which kind of imaging? (Ultrasound)** |  |  | 0.49 |
| Unchecked | 31 (24.8%) | 76 (28.1%) |  |
| Checked | 94 (75.2%) | 194 (71.9%) |  |
| **Which kind of imaging? (X-ray)** |  |  | 0.34 |
| Unchecked | 108 (86.4%) | 223 (82.6%) |  |
| Checked | 17 (13.6%) | 47 (17.4%) |  |
| **Which kind of imaging? (Un-enhanced CT scan)** |  |  | 0.47 |
| Unchecked | 95 (76.0%) | 214 (79.3%) |  |
| Checked | 30 (24.0%) | 56 (20.7%) |  |
| **Which kind of imaging? (Enhanced CT scan)** |  |  | 0.056 |
| Unchecked | 112 (89.6%) | 256 (94.8%) |  |
| Checked | 13 (10.4%) | 14 (5.2%) |  |

**Supplementary Table 3. Nutritional inquiry divided by geographic area**

|  | **non-EU** | **EU** | **p-value** |
| --- | --- | --- | --- |
|  | 125 | 270 |  |
| **Do you perform a nutritional work-up on your stone patients?** |  |  | <0.001 |
| No | 53 (42.4%) | 69 (25.6%) |  |
| Yes | 61 (48.8%) | 184 (68.1%) |  |
| Missing | 11 (8.8%) | 17 (6.3%) |  |
| **Which kind of nutritional work-up? (Diet diary)** |  |  | 0.047 |
| Unchecked | 80 (64.0%) | 144 (53.3%) |  |
| Checked | 45 (36.0%) | 126 (46.7%) |  |
| **Which kind of nutritional work-up? (24h recall)** |  |  | 0.51 |
| Unchecked | 107 (85.6%) | 224 (83.0%) |  |
| Checked | 18 (14.4%) | 46 (17.0%) |  |
| **Which kind of nutritional work-up? (Food-frequency questionnaire (full))** |  |  | 0.60 |
| Unchecked | 115 (92.0%) | 244 (90.4%) |  |
| Checked | 10 (8.0%) | 26 (9.6%) |  |
| **Which kind of nutritional work-up? (Food-frequency questionnaire (screen))** |  |  | 0.29 |
| Unchecked | 115 (92.0%) | 239 (88.5%) |  |
| Checked | 10 (8.0%) | 31 (11.5%) |  |
| **Does your nutritional work-up include evaluation by a dietitian?** |  |  | 0.20 |
| No | 76 (60.8%) | 151 (55.9%) |  |
| Yes | 38 (30.4%) | 102 (37.8%) |  |
| Missing | 11 (8.8%) | 17 (6.3%) |  |

**Supplementary Table 4. Special tests divided for geographic area**

|  | **non-EU** | **EU** | **p-value** |
| --- | --- | --- | --- |
|  | 125 | 270 |  |
| **Do you perform a DXA scan as part of your metabolic work-up?** |  |  | 0.002 |
| Always | 5 (4.0%) | 7 (2.6%) |  |
| Based on selected criteria | 36 (28.8%) | 133 (49.3%) |  |
| Never | 66 (52.8%) | 105 (38.9%) |  |
| Missing | 18 (14.4%) | 25 (9.3%) |  |
| **Which criteria do you follow for DXA scan? (choice=Hypercalciuria)** |  |  | 0.003 |
| Unchecked | 101 (80.8%) | 178 (65.9%) |  |
| Checked | 24 (19.2%) | 92 (34.1%) |  |
| **Which criteria do you follow for DXA scan? (choice=Hypocitraturia)** |  |  | 0.22 |
| Unchecked | 118 (94.4%) | 245 (90.7%) |  |
| Checked | 7 (5.6%) | 25 (9.3%) |  |
| **Which criteria do you follow for DXA scan? (choice=Stone composition)** |  |  | 0.30 |
| Unchecked | 105 (84.0%) | 215 (79.6%) |  |
| Checked | 20 (16.0%) | 55 (20.4%) |  |
| **Do you perform an acidification test in your metabolic work-up?** |  |  | 0.10 |
| Always | 6 (4.8%) | 11 (4.1%) |  |
| Based on selected criteria | 17 (13.6%) | 64 (23.7%) |  |
| Never | 84 (67.2%) | 169 (62.6%) |  |
| Missing | 18 (14.4%) | 26 (9.6%) |  |
| **Which type of acidification test do you perform? (choice= Full ammonium chloride )** |  |  | 0.63 |
| Unchecked | 117 (93.6%) | 249 (92.2%) |  |
| Checked | 8 (6.4%) | 21 (7.8%) |  |
| **Which type of acidification test do you perform? (choice= Modified ammonium chloride )** |  |  | 0.16 |
| Unchecked | 120 (96.0%) | 249 (92.2%) |  |
| Checked | 5 (4.0%) | 21 (7.8%) |  |
| **Which type of acidification test do you perform? (choice=Furosemide/fludrocortistone)** |  |  | 0.051 |
| Unchecked | 117 (93.6%) | 235 (87.0%) |  |
| Checked | 8 (6.4%) | 35 (13.0%) |  |

**Supplementary Table 5. Laboratory investigations divided for geographic area**

|  | **non-EU** | **EU** | **p-value** |
| --- | --- | --- | --- |
|  | 125 | 270 |  |
| **Does your metabolic work-up include laboratory investigations on blood samples?** |  |  | 0.061 |
| In all patients | 72 (57.6%) | 206 (76.3%) |  |
| In selected patients | 16 (12.8%) | 21 (7.8%) |  |
| No | 3 (2.4%) | 4 (1.5%) |  |
| Missing | 34 (27.2%) | 39 (14.4%) |  |
| **Do you perform blood tests:** |  |  | 0.034 |
| Only at baseline | 19 (15.2%) | 25 (9.3%) |  |
| At baseline and follow-up | 68 (54.4%) | 200 (74.1%) |  |
| Only at follow-up | 0 (0.0%) | 2 (0.7%) |  |
| Missing | 38 (30.4%) | 43 (15.9%) |  |
| **Does your metabolic work-up include laboratory investigations on spot urine samples?** |  |  | 0.057 |
| In all patients | 55 (44.0%) | 172 (63.7%) |  |
| In selected patients | 12 (9.6%) | 18 (6.7%) |  |
| No | 23 (18.4%) | 41 (15.2%) |  |
| Missing | 35 (28.0%) | 39 (14.4%) |  |
| **Do you perform spot urine tests:** |  |  | 0.31 |
| Only at baseline | 9 (7.2%) | 16 (5.9%) |  |
| At baseline and follow-up | 53 (42.4%) | 169 (62.6%) |  |
| Only at follow-up | 3 (2.4%) | 5 (1.9%) |  |
| Missing | 60 (48.0%) | 80 (29.6%) |  |
| **Does your metabolic work-up include laboratory investigations on 24h urine samples?** |  |  | 0.003 |
| In all patients | 41 (32.8%) | 156 (57.8%) |  |
| In selected patients | 34 (27.2%) | 54 (20.0%) |  |
| No | 13 (10.4%) | 21 (7.8%) |  |
| Missing | 37 (29.6%) | 39 (14.4%) |  |
| **Do you perform 24h urine tests:** |  |  | 0.085 |
| Only at baseline | 22 (17.6%) | 39 (14.4%) |  |
| At baseline and follow-up | 48 (38.4%) | 162 (60.0%) |  |
| Only at follow-up | 5 (4.0%) | 9 (3.3%) |  |
| Missing | 50 (40.0%) | 60 (22.2%) |  |
| **How many 24h urine collections per work-up do you perform at baseline?** |  |  | 0.008 |
| One | 35 (28.0%) | 126 (46.7%) |  |
| Two | 24 (19.2%) | 66 (24.4%) |  |
| More than two | 10 (8.0%) | 8 (3.0%) |  |
| Missing | 56 (44.8%) | 70 (25.9%) |  |
| **How many 24h urine collections per work-up do you perform at follow-up?** |  |  | 0.064 |
| One | 31 (24.8%) | 128 (47.4%) |  |
| Two | 10 (8.0%) | 22 (8.1%) |  |
| More than two | 12 (9.6%) | 21 (7.8%) |  |
| Missing | 72 (57.6%) | 99 (36.7%) |  |
| **Which kind of 24h urine collection do you perform? (choice=Plain bottle)** |  |  | 0.013 |
| Unchecked | 69 (55.2%) | 113 (41.9%) |  |
| Checked | 56 (44.8%) | 157 (58.1%) |  |
| **Which kind of 24h urine collection do you perform? (choice=Acidified bottle)** |  |  | 0.68 |
| Unchecked | 93 (74.4%) | 206 (76.3%) |  |
| Checked | 32 (25.6%) | 64 (23.7%) |  |
| **Which kind of 24h urine collection do you perform? (choice=Alkalinized bottle)** |  |  | 0.026 |
| Unchecked | 115 (92.0%) | 262 (97.0%) |  |
| Checked | 10 (8.0%) | 8 (3.0%) |  |
| **Which kind of 24h urine collection do you perform? (choice=Bottle with antibacterial)** |  |  | 0.28 |
| Unchecked | 119 (95.2%) | 249 (92.2%) |  |
| Checked | 6 (4.8%) | 21 (7.8%) |  |
| **Do you routinely obtain supersaturation indices from 24h urine collections?** |  |  | 0.10 |
| No | 73 (58.4%) | 192 (71.1%) |  |
| Yes | 2 (1.6%) | 17 (6.3%) |  |
| Missing | 50 (40.0%) | 61 (22.6%) |  |
| **Which supersaturation indices do you obtain? (choice=Activity products)** |  |  | 0.33 |
| Unchecked | 123 (98.4%) | 261 (96.7%) |  |
| Checked | 2 (1.6%) | 9 (3.3%) |  |
| **Which supersaturation indices do you obtain? (choice=EQUIL-2)** |  |  | 0.24 |
| Unchecked | 125 (100.0%) | 267 (98.9%) |  |
| Checked | 0 (0.0%) | 3 (1.1%) |  |
| **Which supersaturation indices do you obtain? (choice=JESS)** |  |  |  |
| Unchecked | 125 (100.0%) | 270 (100.0%) |  |
| **Which supersaturation indices do you obtain? (choice=PSF)** |  |  | 0.50 |
| Unchecked | 125 (100.0%) | 269 (99.6%) |  |
| Checked | 0 (0.0%) | 1 (0.4%) |  |
| **Which supersaturation indices do you obtain? (choice=Betas)** |  |  |  |
| Unchecked | 125 (100.0%) | 270 (100.0%) |  |
| **Do you routinely obtain stone composition analysis?** |  |  | <0.001 |
| No | 41 (32.8%) | 58 (21.5%) |  |
| Yes | 53 (42.4%) | 176 (65.2%) |  |
| Missing | 31 (24.8%) | 36 (13.3%) |  |
| **Which kind of stone composition analysis? (choice=IR-spectroscopy)** |  |  | <0.001 |
| Unchecked | 112 (89.6%) | 195 (72.2%) |  |
| Checked | 13 (10.4%) | 75 (27.8%) |  |
| **Which kind of stone composition analysis? (choice=X-ray diffraction)** |  |  | 0.68 |
| Unchecked | 115 (92.0%) | 245 (90.7%) |  |
| Checked | 10 (8.0%) | 25 (9.3%) |  |
| **Which kind of stone composition analysis? (choice=Chemical analysis)** |  |  | 0.28 |
| Unchecked | 83 (66.4%) | 164 (60.7%) |  |
| Checked | 42 (33.6%) | 106 (39.3%) |  |

**Supplementary Table 6. Blood laboratory investigations**

|  | **Value** |
| --- | --- |
|  | 395 |
| **Which of these blood parameters are included in your metabolic work-up? (choice= Creatinine)** |  |
| Unchecked | 84 (21.3%) |
| Checked | 311 (78.7%) |
| **Which of these blood parameters are included in your metabolic work-up? (choice= Urea)** |  |
| Unchecked | 120 (30.4%) |
| Checked | 275 (69.6%) |
| **Which of these blood parameters are included in your metabolic work-up? (choice= Sodium)** |  |
| Unchecked | 99 (25.1%) |
| Checked | 296 (74.9%) |
| **Which of these blood parameters are included in your metabolic work-up? (choice= Potassium)** |  |
| Unchecked | 102 (25.8%) |
| Checked | 293 (74.2%) |
| **Which of these blood parameters are included in your metabolic work-up? (choice= Calcium)** |  |
| Unchecked | 89 (22.5%) |
| Checked | 306 (77.5%) |
| **Which of these blood parameters are included in your metabolic work-up? (choice= Phosphorus)** |  |
| Unchecked | 97 (24.6%) |
| Checked | 298 (75.4%) |
| **Which of these blood parameters are included in your metabolic work-up? (choice= Magnesium)** |  |
| Unchecked | 146 (37.0%) |
| Checked | 249 (63.0%) |
| **Which of these blood parameters are included in your metabolic work-up? (choice= Uric Acid)** |  |
| Unchecked | 89 (22.5%) |
| Checked | 306 (77.5%) |
| **Which of these blood parameters are included in your metabolic work-up? (choice= Chloride)** |  |
| Unchecked | 181 (45.8%) |
| Checked | 214 (54.2%) |
| **Which of these blood parameters are included in your metabolic work-up? (choice= pH)** |  |
| Unchecked | 156 (39.5%) |
| Checked | 239 (60.5%) |
| **Which of these blood parameters are included in your metabolic work-up? (choice= Bicarbonate)** |  |
| Unchecked | 138 (34.9%) |
| Checked | 257 (65.1%) |
| **Which of these blood parameters are included in your metabolic work-up? (choice= PTH)** |  |
| Unchecked | 111 (28.1%) |
| Checked | 284 (71.9%) |
| **Which of these blood parameters are included in your metabolic work-up? (choice= 25(OH) vitamin D)** |  |
| Unchecked | 188 (47.6%) |
| Checked | 207 (52.4%) |
| **Which of these blood parameters are included in your metabolic work-up? (choice= 1,25(OH)-2 vitamin D)** |  |
| Unchecked | 283 (71.6%) |
| Checked | 112 (28.4%) |

**Supplementary Table 7. Spot urine laboratory investigations**

|  | **Values** |
| --- | --- |
|  | 395 |
| **Which of these spot urine parameters are included in your metabolic work-up? (choice= Creatinine)** |  |
| Unchecked | 213 (53.9%) |
| Checked | 182 (46.1%) |
| **Which of these spot urine parameters are included in your metabolic work-up? (choice= Urea)** |  |
| Unchecked | 296 (74.9%) |
| Checked | 99 (25.1%) |
| **Which of these spot urine parameters are included in your metabolic work-up? (choice= Sodium)** |  |
| Unchecked | 249 (63.0%) |
| Checked | 146 (37.0%) |
| **Which of these spot urine parameters are included in your metabolic work-up? (choice= Potassium)** |  |
| Unchecked | 275 (69.6%) |
| Checked | 120 (30.4%) |
| **Which of these spot urine parameters are included in your metabolic work-up? (choice= Calcium)** |  |
| Unchecked | 211 (53.4%) |
| Checked | 184 (46.6%) |
| **Which of these spot urine parameters are included in your metabolic work-up? (choice= Phosphorus)** |  |
| Unchecked | 248 (62.8%) |
| Checked | 147 (37.2%) |
| **Which of these spot urine parameters are included in your metabolic work-up? (choice= Magnesium)** |  |
| Unchecked | 277 (70.1%) |
| Checked | 118 (29.9%) |
| **Which of these spot urine parameters are included in your metabolic work-up? (choice= Uric Acid)** |  |
| Unchecked | 239 (60.5%) |
| Checked | 156 (39.5%) |
| **Which of these spot urine parameters are included in your metabolic work-up? (choice= Chloride)** |  |
| Unchecked | 319 (80.8%) |
| Checked | 76 (19.2%) |
| **Which of these spot urine parameters are included in your metabolic work-up? (choice= Oxalate)** |  |
| Unchecked | 242 (61.3%) |
| Checked | 153 (38.7%) |
| **Which of these spot urine parameters are included in your metabolic work-up? (choice= Citrate)** |  |
| Unchecked | 258 (65.3%) |
| Checked | 137 (34.7%) |
| **Which of these spot urine parameters are included in your metabolic work-up? (choice= Cystine)** |  |
| Unchecked | 291 (73.7%) |
| Checked | 104 (26.3%) |
| **Which of these spot urine parameters are included in your metabolic work-up? (choice= Ammonium)** |  |
| Unchecked | 369 (93.4%) |
| Checked | 26 (6.6%) |
| **Which of these spot urine parameters are included in your metabolic work-up? (choice= Sulfate)** |  |
| Unchecked | 380 (96.2%) |
| Checked | 15 (3.8%) |
| **Which of these spot urine parameters are included in your metabolic work-up? (choice= pH)** |  |
| Unchecked | 188 (47.6%) |
| Checked | 207 (52.4%) |
| **Which of these spot urine parameters are included in your metabolic work-up? (choice= Bicarbonate)** |  |
| Unchecked | 331 (83.8%) |
| Checked | 64 (16.2%) |
| **Which of these spot urine parameters are included in your metabolic work-up? (choice= Physico-chemical examination)** |  |
| Unchecked | 291 (73.7%) |
| Checked | 104 (26.3%) |
| **Which of these spot urine parameters are included in your metabolic work-up? (choice= Sediment examination)** |  |
| Unchecked | 197 (49.9%) |
| Checked | 198 (50.1%) |
| **Which of these spot urine parameters are included in your metabolic work-up? (choice= Urine culture)** |  |
| Unchecked | 214 (54.2%) |
| Checked | 181 (45.8%) |

**Supplementary Table 8. 24h urine laboratory investigations**

| **Which of these 24h urine parameters are included in your metabolic work-up? (choice= Creatinine)** |  |
| --- | --- |
| Unchecked | 154 (39.0%) |
| Checked | 241 (61.0%) |
| **Which of these 24h urine parameters are included in your metabolic work-up? (choice= Urea)** |  |
| Unchecked | 248 (62.8%) |
| Checked | 147 (37.2%) |
| **Which of these 24h urine parameters are included in your metabolic work-up? (choice= Sodium)** |  |
| Unchecked | 169 (42.8%) |
| Checked | 226 (57.2%) |
| **Which of these 24h urine parameters are included in your metabolic work-up? (choice= Potassium)** |  |
| Unchecked | 207 (52.4%) |
| Checked | 188 (47.6%) |
| **Which of these 24h urine parameters are included in your metabolic work-up? (choice= Calcium)** |  |
| Unchecked | 123 (31.1%) |
| Checked | 272 (68.9%) |
| **Which of these 24h urine parameters are included in your metabolic work-up? (choice= Phosphorus)** |  |
| Unchecked | 170 (43.0%) |
| Checked | 225 (57.0%) |
| **Which of these 24h urine parameters are included in your metabolic work-up? (choice= Magnesium)** |  |
| Unchecked | 211 (53.4%) |
| Checked | 184 (46.6%) |
| **Which of these 24h urine parameters are included in your metabolic work-up? (choice= Uric Acid)** |  |
| Unchecked | 146 (37.0%) |
| Checked | 249 (63.0%) |
| **Which of these 24h urine parameters are included in your metabolic work-up? (choice= Chloride)** |  |
| Unchecked | 291 (73.7%) |
| Checked | 104 (26.3%) |
| **Which of these 24h urine parameters are included in your metabolic work-up? (choice= Oxalate)** |  |
| Unchecked | 152 (38.5%) |
| Checked | 243 (61.5%) |
| **Which of these 24h urine parameters are included in your metabolic work-up? (choice= Citrate)** |  |
| Unchecked | 164 (41.5%) |
| Checked | 231 (58.5%) |
| **Which of these 24h urine parameters are included in your metabolic work-up? (choice= Cystine)** |  |
| Unchecked | 267 (67.6%) |
| Checked | 128 (32.4%) |
| **Which of these 24h urine parameters are included in your metabolic work-up? (choice= Ammonium)** |  |
| Unchecked | 358 (90.6%) |
| Checked | 37 (9.4%) |
| **Which of these 24h urine parameters are included in your metabolic work-up? (choice= Sulfate)** |  |
| Unchecked | 375 (94.9%) |
| Checked | 20 (5.1%) |
| **Which of these 24h urine parameters are included in your metabolic work-up? (choice= pH)** |  |
| Unchecked | 250 (63.3%) |
| Checked | 145 (36.7%) |
| **Which of these 24h urine parameters are included in your metabolic work-up? (choice= Bicarbonate)** |  |
| Unchecked | 333 (84.3%) |
| Checked | 62 (15.7%) |

**Supplementary Table 9. Demographics characteristics**

| **Which of the following DEMOGRAPHICS data do you collect? (choice=Date of birth)** |  |
| --- | --- |
| Unchecked | 68 (17.2%) |
| Checked | 327 (82.8%) |
| **Which of the following DEMOGRAPHICS data do you collect? (choice=Gender)** |  |
| Unchecked | 61 (15.4%) |
| Checked | 334 (84.6%) |
| **Which of the following DEMOGRAPHICS data do you collect? (choice=Race/ethnicity)** |  |
| Unchecked | 146 (37.0%) |
| Checked | 249 (63.0%) |
| **Which of the following DEMOGRAPHICS data do you collect? (choice=Profession)** |  |
| Unchecked | 213 (53.9%) |
| Checked | 182 (46.1%) |
| **Which of the following DEMOGRAPHICS data do you collect? (choice=Education level)** |  |
| Unchecked | 263 (66.6%) |
| Checked | 132 (33.4%) |
| **Which of the following DEMOGRAPHICS data do you collect? (choice=Income level)** |  |
| Unchecked | 337 (85.3%) |
| Checked | 58 (14.7%) |

**Supplementary Table 10. General medical status**

| **Which of the following MEDICAL STATUS information do you collect: (choice=Hyperparathyroidism)** |  |
| --- | --- |
| Unchecked | 84 (21.3%) |
| Checked | 311 (78.7%) |
| **Which of the following MEDICAL STATUS information do you collect: (choice=Hyperthyroidism)** |  |
| Unchecked | 207 (52.4%) |
| Checked | 188 (47.6%) |
| **Which of the following MEDICAL STATUS information do you collect: (choice=Sarcoidosis)** |  |
| Unchecked | 244 (61.8%) |
| Checked | 151 (38.2%) |
| **Which of the following MEDICAL STATUS information do you collect: (choice=Vitamin D excess)** |  |
| Unchecked | 120 (30.4%) |
| Checked | 275 (69.6%) |
| **Which of the following MEDICAL STATUS information do you collect: (choice=Calcium supplements)** |  |
| Unchecked | 89 (22.5%) |
| Checked | 306 (77.5%) |
| **Which of the following MEDICAL STATUS information do you collect: (choice=Prolonged immobilization)** |  |
| Unchecked | 149 (37.7%) |
| Checked | 246 (62.3%) |
| **Which of the following MEDICAL STATUS information do you collect: (choice=Clinical evidence of bone disease)** |  |
| Unchecked | 155 (39.2%) |
| Checked | 240 (60.8%) |
| **Which of the following MEDICAL STATUS information do you collect: (choice=Malignant neoplasms)** |  |
| Unchecked | 201 (50.9%) |
| Checked | 194 (49.1%) |
| **Which of the following MEDICAL STATUS information do you collect: (choice=Genetic conditions)** |  |
| Unchecked | 144 (36.5%) |
| Checked | 251 (63.5%) |
| **Which of the following MEDICAL STATUS information do you collect: (choice=Medullary sponge kidney)** |  |
| Unchecked | 167 (42.3%) |
| Checked | 228 (57.7%) |
| **Which of the following MEDICAL STATUS information do you collect: (choice=Enteric hyperoxaluria)** |  |
| Unchecked | 185 (46.8%) |
| Checked | 210 (53.2%) |
| **Which of the following MEDICAL STATUS information do you collect: (choice=Bowel disease)** |  |
| Unchecked | 178 (45.1%) |
| Checked | 217 (54.9%) |
| **Which of the following MEDICAL STATUS information do you collect: (choice=Chronic pancreatitis)** |  |
| Unchecked | 268 (67.8%) |
| Checked | 127 (32.2%) |
| **Which of the following MEDICAL STATUS information do you collect: (choice=Vitamin C supplements)** |  |
| Unchecked | 198 (50.1%) |
| Checked | 197 (49.9%) |
| **Which of the following MEDICAL STATUS information do you collect: (choice=Chronic diarrhea)** |  |
| Unchecked | 204 (51.6%) |
| Checked | 191 (48.4%) |
| **Which of the following MEDICAL STATUS information do you collect: (choice=Lithogenic drugs)** |  |
| Unchecked | 171 (43.3%) |
| Checked | 224 (56.7%) |
| **Which of the following MEDICAL STATUS information do you collect: (choice=Urinary infections)** |  |
| Unchecked | 81 (20.5%) |
| Checked | 314 (79.5%) |
| **Which of the following MEDICAL STATUS information do you collect: (choice=Gouty diathesis)** |  |
| Unchecked | 172 (43.5%) |
| Checked | 223 (56.5%) |
| **Which of the following MEDICAL STATUS information do you collect: (choice=Diabetes)** |  |
| Unchecked | 161 (40.8%) |
| Checked | 234 (59.2%) |
| **Which of the following MEDICAL STATUS information do you collect: (choice=High blood pressure)** |  |
| Unchecked | 124 (31.4%) |
| Checked | 271 (68.6%) |
| **Which of the following MEDICAL STATUS information do you collect: (choice=Dyslipidemia)** |  |
| Unchecked | 210 (53.2%) |
| Checked | 185 (46.8%) |
| **Which of the following MEDICAL STATUS information do you collect: (choice=Single kidney)** |  |
| Unchecked | 120 (30.4%) |
| Checked | 275 (69.6%) |
| **Which of the following MEDICAL STATUS information do you collect: (choice=Chronic kidney disease)** |  |
| Unchecked | 83 (21.0%) |
| Checked | 312 (79.0%) |

**Supplementary Table 11. Kidney stone history**

| **Which of the following STONE HISTORY data do you collect: (choice=Date of first stone event)** |  |
| --- | --- |
| Unchecked | 77 (19.5%) |
| Checked | 318 (80.5%) |
| **Which of the following STONE HISTORY data do you collect: (choice=Number of previous events)** |  |
| Unchecked | 83 (21.0%) |
| Checked | 312 (79.0%) |
| **Which of the following STONE HISTORY data do you collect: (choice=List of urological procedures)** |  |
| Unchecked | 84 (21.3%) |
| Checked | 311 (78.7%) |
| **Which of the following STONE HISTORY data do you collect: (choice=Monolateral/bilateral disease)** |  |
| Unchecked | 86 (21.8%) |
| Checked | 309 (78.2%) |
| **Which of the following STONE HISTORY data do you collect: (choice=Recurrent disease)** |  |
| Unchecked | 75 (19.0%) |
| Checked | 320 (81.0%) |
| **Which of the following STONE HISTORY data do you collect: (choice=Family history of stones)** |  |
| Unchecked | 70 (17.7%) |
| Checked | 325 (82.3%) |

**Supplementary Table 12. Kidney stone status**

| **Which of the following STONE STATUS information do you collect: (choice=Date of imaging)** |  |
| --- | --- |
| Unchecked | 101 (25.6%) |
| Checked | 294 (74.4%) |
| **Which of the following STONE STATUS information do you collect: (choice=Type of imaging)** |  |
| Unchecked | 89 (22.5%) |
| Checked | 306 (77.5%) |
| **Which of the following STONE STATUS information do you collect: (choice=Number of stones)** |  |
| Unchecked | 97 (24.6%) |
| Checked | 298 (75.4%) |
| **Which of the following STONE STATUS information do you collect: (choice=Laterality of stones)** |  |
| Unchecked | 115 (29.1%) |
| Checked | 280 (70.9%) |
| **Which of the following STONE STATUS information do you collect: (choice=Size of stones)** |  |
| Unchecked | 103 (26.1%) |
| Checked | 292 (73.9%) |
| **Which of the following STONE STATUS information do you collect: (choice=Presence of nephrocalcinosis)** |  |
| Unchecked | 94 (23.8%) |
| Checked | 301 (76.2%) |

**Supplementary Table 13. Diet and lifestyle**

| **Which of the following DIET/LIFESTYLE information do you collect: (choice=Amount of fluids)** |  |
| --- | --- |
| Unchecked | 91 (23.0%) |
| Checked | 304 (77.0%) |
| **Which of the following DIET/LIFESTYLE information do you collect: (choice=Type of fluids)** |  |
| Unchecked | 146 (37.0%) |
| Checked | 249 (63.0%) |
| **Which of the following DIET/LIFESTYLE information do you collect: (choice=Intake of calcium)** |  |
| Unchecked | 147 (37.2%) |
| Checked | 248 (62.8%) |
| **Which of the following DIET/LIFESTYLE information do you collect: (choice=Intake of sodium)** |  |
| Unchecked | 110 (27.8%) |
| Checked | 285 (72.2%) |
| **Which of the following DIET/LIFESTYLE information do you collect: (choice=Intake of animal protein)** |  |
| Unchecked | 154 (39.0%) |
| Checked | 241 (61.0%) |
| **Which of the following DIET/LIFESTYLE information do you collect: (choice=Intake of fruits/vegetables)** |  |
| Unchecked | 192 (48.6%) |
| Checked | 203 (51.4%) |
| **Which of the following DIET/LIFESTYLE information do you collect: (choice=Intake of oxalate)** |  |
| Unchecked | 194 (49.1%) |
| Checked | 201 (50.9%) |
| **Which of the following DIET/LIFESTYLE information do you collect: (choice=Intake of fructose)** |  |
| Unchecked | 314 (79.5%) |
| Checked | 81 (20.5%) |
| **Which of the following DIET/LIFESTYLE information do you collect: (choice=Total calories)** |  |
| Unchecked | 303 (76.7%) |
| Checked | 92 (23.3%) |
| **Which of the following DIET/LIFESTYLE information do you collect: (choice=Physical activity)** |  |
| Unchecked | 163 (41.3%) |
| Checked | 232 (58.7%) |
| **Which of the following DIET/LIFESTYLE information do you collect: (choice=Smoking status)** |  |
| Unchecked | 198 (50.1%) |
| Checked | 197 (49.9%) |
| **Which of the following DIET/LIFESTYLE information do you collect: (choice=Alcohol consumption)** |  |
| Unchecked | 223 (56.5%) |
| Checked | 172 (43.5%) |
| **Which of the following DIET/LIFESTYLE information do you collect: (choice=Quality of life)** |  |
| Unchecked | 287 (72.7%) |
| Checked | 108 (27.3%) |

**Supplementary Table 14. Physical examination**

| **Which of the following PHYSICAL EXAMINATION data do you collect: (choice=Height)** |  |
| --- | --- |
| Unchecked | 118 (29.9%) |
| Checked | 277 (70.1%) |
| **Which of the following PHYSICAL EXAMINATION data do you collect: (choice=Weight)** |  |
| Unchecked | 91 (23.0%) |
| Checked | 304 (77.0%) |
| **Which of the following PHYSICAL EXAMINATION data do you collect: (choice=Waist circumference)** |  |
| Unchecked | 338 (85.6%) |
| Checked | 57 (14.4%) |
| **Which of the following PHYSICAL EXAMINATION data do you collect: (choice=Blood pressure)** |  |
| Unchecked | 84 (21.3%) |
| Checked | 311 (78.7%) |

**Supplementary Table 15. Administrative/research tools**

| **Do you use questionnaires to obtain information from your stone patients?** |  |
| --- | --- |
| No | 308 (78.0%) |
| Yes | 52 (13.2%) |
| Missing | 35 (8.9%) |
| **Which kind of questionnaire do you use? (Self-made, not validated)** |  |
| No | 354 (89.6%) |
| Yes | 41 (10.4%) |
| **Which kind of questionnaire do you use? (Self-made, validated)** |  |
| No | 387 (98.0%) |
| Yes | 8 (2.0%) |
| **Which kind of questionnaire do you use? (Not self-made, validated)** |  |
| No | 389 (98.5%) |
| Yes | 6 (1.5%) |
| **Do you collect information on your stone patients: Through an Institution** |  |
| No | 213 (53.9%) |
| Yes | 182 (46.1%) |
| **Do you collect information on your stone patients: Through other electron forms** |  |
| No | 336 (85.1%) |
| Yes | 59 (14.9%) |
| **Do you collect information on your stone patients: On paper forms** |  |
| No | 250 (63.3%) |
| Yes | 145 (36.7%) |
| **Do you keep a biobank of your stone patients?** |  |
| No | 311 (78.7%) |
| Yes | 35 (8.9%) |
| Missing | 49 (12.4%) |
| **Which biological samples are stored in your biobank? (Whole blood/DNA)** |  |
| No | 379 (95.9%) |
| Yes | 16 (4.1%) |
| **Which biological samples are stored in your biobank? (Serum/plasma)** |  |
| No | 378 (95.7%) |
| Yes | 17 (4.3%) |
| **Which biological samples are stored in your biobank? (Spot urine)** |  |
| No | 381 (96.5%) |
| Yes | 14 (3.5%) |
| **Which biological samples are stored in your biobank? (24h urine)** |  |
| No | 379 (95.9%) |
| Yes | 16 (4.1%) |

**Supplementary Table 16. Incident stone event**

| **How do you define an incident stone event? (choice=New stone formation)** |  |  | 0.012 |
| --- | --- | --- | --- |
| Unchecked | 49 (39.2%) | 72 (26.7%) |  |
| Checked | 76 (60.8%) | 198 (73.3%) |  |
| **How do you define an incident stone event? (choice=Growth of a previous stone)** |  |  | 1.00 |
| Unchecked | 81 (64.8%) | 175 (64.8%) |  |
| Checked | 44 (35.2%) | 95 (35.2%) |  |
| **How do you define an incident stone event? (choice=Stone expulsion)** |  |  | 0.25 |
| Unchecked | 78 (62.4%) | 152 (56.3%) |  |
| Checked | 47 (37.6%) | 118 (43.7%) |  |
| **How do you define an incident stone event? (choice=Urological intervention)** |  |  | 0.077 |
| Unchecked | 83 (66.4%) | 154 (57.0%) |  |
| Checked | 42 (33.6%) | 116 (43.0%) |  |
| **How do you define an incident stone event? (choice=Renal colic)** |  |  | 0.026 |
| Unchecked | 72 (57.6%) | 123 (45.6%) |  |
| Checked | 53 (42.4%) | 147 (54.4%) |  |

**Supplementary Table 17. Overall score items**

| **Item** | **Points** |
| --- | --- |
| Formal referral criteria | 1 |
| Formal follow-up scheme | 1 |
| Systematic imaging | 1 |
| Nutritional workup | 1 |
| Dietitian available | 1 |
| Data collection with electronic forms | 1 |
| Use of validated questionnaires | 1 |
| Performs DEXA | 1 |
| Performs acidification test | 1 |
| Biobanking | 1 |
| Optimal stone composition analysis | 1 |
| Number of items reported for stone history/comorbidities/medications/lab | 1 Q4  2 Q3  3 Q2  4 Q1 |
| Total score range | 1 - 15 |
