# Supplementary material for: Practice patterns of kidney stone management across European and non-European centers: an in-depth investigation from the European Renal Stone Network (ERSN)
Source: J Nephrol. 2020 Sep 12;34(4):1337–46. doi: 10.1007/s40620-020-00854-6 (PMC8357688; doi:10.1007/s40620-020-00854-6)
Supplement: Supplementary file 2 — Supplementary file2 (DOCX 24 kb) [file 40620_2020_854_MOESM2_ESM.docx]

**Acknowledgments list**

Adbi Haloob Imad (Bathurst Hospital. NSW 2795. Australia), Ağbaş Ayşe (University of Health Sciences, Haseki Education and Research Hospital, Pediatric Nephrology, Istanbul, Turkey), Ahmed Saeed (South Tyneside Sunderland Foundation Trust, Sunderland, Tyne and Wear, UK), Akram Eid Loai (Pediatric Nephrology Department, Dubai Hospital, Dubai – UAE), Aksenova Marina (Y.Veltischev Research and Clinical Institute for Pediatrics at N.Pirogov Russian National Research Medical University, Moscow), Amair Miani Pablo (Hospital de Clìnicas Caracas, Venezuela), Ariceta Gema (Nefrología Pediátrica Hospital Universitari Vall d' Hebron Barcelona, Spain), Arroyo David (Nephrology Department. Hospital General Universitario Gregorio Marañón, Madrid, Spain), Arrojo Alonso Fernanda (Hospital Arquitecto Marcide Ferrol), Arzoz-Fàbregas Montserrat (Urology Department, Hospital Germans Trias I Pujol. Badalona. Barcelona), Ashraf Junaid (Leeds General Infirmary, Leeds, UK), Auñón Pilar (Nephrology Department Hospital Doce de Octubre, Madrid, Spain), Avellaneda Enrique Cao (Complejo Hospitalario Universitario de Cartagena Spain), Azharuddin Mohammed (Department of Nephrology and Kidney Transplantation, Armed Forces Hospital Taif Region, Alhada, Kingdomnof Saudi Arabia), Balan Satish Kerala (Institute of Medical Sciences, Trivandrum Kerala India), Barbarini Silvia, Università Cattolica del Sacro Cuore, Roma, Italia), Baquero Rodriguez Richard (San Vicente University Hospital - Antioquia University, Medicine Faculty, Medellín, Colombia), Barra Tiago Luís Correia (Serviço de Nefrologia, Centro Hospitalar Tondela Viseu, Viseu, Portugal), Bekiroğlu Yılmaz Ebru (Dr Behçet Uz Children's Hospital İzmir Turkey), Belguesmia Zaineb (Mgen Clinique, Maisons Lafitte, France), Bellizzi Vincenzo (Nephrology and Dialysis Division, University Hospital “San Giovanni di Dio e Ruggi d’Aragona”, Salerno – Italy), Beringer Ortraud (Department of Pediatrics, University Medical Center Ulm), Biswanath Basu (Division of Pediatric Nephrology, Department of Pediatrics, Nilratan Sircar Medical College & Hospital, Kolkata), Bordasch Huppes Schneider Luciane (Hospital Infantil Joana de Gusmão, Florianópolis, SC, Brazil), Borovitz Yael (Nephrology institute, Schneider Children's Medical Center, Petah Tikva, Israel), Braconnier Philippe (Division of Nephrology and Hypertension, Lausanne University Hospital and University of Lausanne, Lausanne Switzerland), Brulez Harald F.H. (OLVG hospital, Amsterdam The Netherlands), Buades Fuster Juan Manuel (Nephrology Department, Hospital Universitario Son Llàtzer, Majorca, Spain), Bulum Burcu Akbulut (Acıbadem Mehmet Ali Aydınlar University, School of Medicine Department of Pediatric Nephrology), Büttner Stefan (Medical Clinic III, Department of Nephrology, University Hospital Frankfurt, Frankfurt am Main, Germany), Caeiro Fernando (Hospital Curry Cabral, Lisboa), Calaud Frederic (Department of Nephrology , King Faisan Specialist Hospital &Research Center, Jeddah KSA), Calimeri Sebastiano (Unit of Nephrology, Department of Clinical and Experimental Medicine, University of Messina), Campbell Helen (North Bristol NHS Trust, UK), Cansino Ramon (Section Chair of Endourology and Stone Disease. La Paz University Hospital. Madrid. Spain), Capolongo Giovanna (Department of Translational Medical Sciences, University of, Campania “Luigi Vanvitelli”, 80131 Naples, Italy), Catalano Concetta (Nephrology, Dialysis and Transplantation Unit Hopital Erasme Bruxelles Belgium), Chauveau Philippe (Aurad-Aquitaine, Bordeaux, France), Chimenz Roberto (U.O. Pediatric Nephrology with dialysis - A.O.U. G. Martino - Messina Italy), Cleper Roxana Dana Dweq (Children's Hospital, Tel Aviv Sourasky Medical Center, Sackler Medical School, Tel Aviv University. Tel Aviv. Israel), Colussi Giacomo (Division of Nephrology, Dialysis and Renal, Transplantation, ASST GOM Niguarda, Milan, Italy), Conti Giovanni (Pediatric Nephrology Unit, AOU Policlinic G Martino, Messina, Italy), Dalili Nooshin (Labbafinejad Medical Center, CKDRC,Shahid Beheshti University of Medical Sciences, Tehran ,Iran), De Ferrari Maria Elisabetta (Nefrologia, Ospedale Niguarda, Milano, Italy), De Lima Serra Maria Adelaide (Serviço de Nefrologia do Hospital Professor Doutor Fernando Fonseca), De Oliveira Bello Vilber Antonio (Centor Brasiliense de Nefrologia & Diálise), Dinçel Nida Temizkan (Dr Behçet Uz Children's Hospital İzmir Turkey), Dursun Ismail, Erciyes University, Faculty of Medicine, Department of Pediatrics, Division of Nephrology, Kayseri, Turkey), Eleftheriadis Theodoros (Department of Nephrology, Faculty of Medicine, University of Thessaly, Larissa, Greece), Elrggal Mohamed (Kidney and Urology Center), EL Zorkany Khaled Mohammed Amin (Menoufia University, Egypt. Assistant professor in King Faisal University, KSA), Estrade Vincent (Urology dept of CHU Bordeaux, place Amélie Raba Leon, 33000 Bordeaux), Fabris Antonia (UOC Nefrologia, Azienda Ospedaliera Universitaria Integrata di Verona, Verona, Italy), Falbo Enrica (Valiant Clinic, Dubai, UAE), Figueres Lucile (ITUN, CHU de Nantes, France, Université de Nantes), Fonseca Nuno Moreira (Nephrology Department, Central Lisbon University Hospital Center, Lisbon, Portugal), Fontana Francesco (Nephrology and Dialysis Unit, University Hospital of Modena, Modena, Italy), Finlay Eric (Leeds Teaching Hospitals NHS Trust), Fragkidis Stylianos (Nephrology Department, G.H. "G. Papanikolaou", Thessaloniki, Greece), Frajewicki Victor (Department of Nephrology and Hypertension, Carmel Medical Center, Haifa, Israel), Francisco Telma Sofia Carvalho (Unidade de Nefrologia Pediátrica, Hospital de Dona Estefania, Centro Hospitalar e Universitário de Lisboa Central), Frangou Eleni (Department of Nephrology, Limassol General Hospital, Limassol, Cyprus), Garozzo Maurizio (Azienda Sanitaria Provinciale di Catania Ospedale Santa Marta e Santa Venera U.O.C. Nefrologia e Dialisi 95024 Acireale CT Sicilia), Gokce Ibrahim (Marmara University, School of Medicine, Division of Pediatric Nephrology), Grandtnerova Barbara (Zeleny sen Hospital, Banska Bystrica, Slovak Republic), Greenbaum Larry A (Emory University, Atlanta, GA, USA), Guarnieri Andrea (Nephrology Dialysis and Transplant Unit AOU Senese, Italy), Haloob Imad Adbi (1. Bathurst Hospital, NSW Australia. 2. Westmead Hospital, NSW Australia), Helin Karri H. (Section of internal Medicine, Seinäjoki Central Hospital Finland), Hilbrands Luuk (Department of Nephrology, Radboud university medical center, Nijmegen, The Netherlands), Ireneusz Habura Kierownik Oddziału (Nephrology Unit.Szpital Uniwersytecki im.Karola Marcinkowskiego w Zielonej Górze, 65-001 Zielona Góra.Polska), Jobs Katarzyna (Military Institute of Medicine, Pediatric Nephrology Department, Warsaw, Poland), Johnstone Lilian (Department of nephrology, Monash Children’s Hospital, Monash Health, Australia; Department of Paediatrics, Monash University, Melbourne, Australia), Keil Alexander (Nephrologisches Zentrum Villingen- Schwenningen), Kes Petar (Department of Nephrology, Hypertension, Dialysis and Kidney Transplantation University Hospital Centre Zagreb, Zagreb, Croatia), Kuhlmann Martin K (Vivantes Klinikum im Friedrichshain, Berlin, Germany), Kumar Gurinder (Division of Pediatric Nephrology at Sheikh Khalifa Medical City, Abu Dhabi, United Arab Emirates), Lale Sever Fatma (Istanbul University- Cerrahpasa School of Medicine, Turkey), Lambie Mark (Keele University), Lamy Thomas (Nephrology unit, Medipôle Hospital, Noumea, New Caledonia), Langman Craig B (Feinberg School of Medicine, Northwestern University Head, Kidney Diseases, The Ann and Robert H Lurie Children's Hospital of Chicago), Laranjinha Ivo (Hospital de Santa Cruz - Centro Hospitalar de Lisboa Ocidental, Lisbon, Portugal), Lemoine Sandrine (Edouard Herriot Hospital, Hospices civils de Lyon, nephrology unit, Lyon, France), Liakopoulos Vassilios (Division of Nephrology and Hypertension , 1st Department of Internal Medicine, AHEPA Hospital Medical School, Aristotle University of Thessaloniki, Thessaloniki, Greece), Liangos Orfeas (Klinikum Coburg, Germany), Liccardo Amelia (U.O. di Nefrologia e Dialisi, Ospedale Multimedica, Castellanza Varese), Longo Germana (AOU Padua - Pediatric Nephrology Unit), Lopez da Conceição dos Santos Noélis Isabel (Centro Hospitalar Universitário Lisboa Norte, Portugal), Lumbreras Javier (Pediatric Nephrology Unit. University Hospital Son Espases - Balearic Islands Health Research Institute (IdISBa). Palma de Mallorca. Spain), Lungu Adrian Catalin (Fundeni Clinical Institute, Pediatric Nephrology Department, Bucharest, Romania), Maranghini Silvio (Department of Pediatrics, Istituto Mediterraneo per i Trapianti e Terapie ad Alta Specializzazione), Mejía Natalia (School of Medicine- Universidad de Los Andes- Bogotá, Colombia), Mirioglu Safak (Istanbul University Istanbul Faculty of Medicine), Moriconi Diego (Department of Clinical and Experimental Medicine, University of Pisa), Nagra Arvind (Southampton Children's Hospital, Soutbampton, UK), Neto Miguel Moyses (Division of Nephrology, Ribeirao Preto Medical School, Sao Paulo University), Ogrizovic Sanja Simic (General Hospital Medigroup, Belgrade, Serbia), Papasotiriou Mario (Department of Nephrology, University Hospital of Patras, Rion, 26504), Paripovic Dusan (University Children's Hospital, Belgrade, Serbia), Paunic Zoran (Special hospital for hemodialysis "Fresenius Medical Care" Belgrade), Penmatsa Krishnam Raju (OneLife Kidney and Blood Pressure Clinic, Visakhapatnam, India), Pereira Andrè Barreto (Marieta Konder Bornhausen Hospital and Maternity, Itajaí, SC, Brazil), Petkova Kremena (Military Medical Academy, Department of Urology and Nephrology, Georgi Sofiisky blvd. Sofia 1606 Bulgaria), Pleros, Christos (Intensive Care Unit - Chania General Hospital), Prikhodina Larina (Division of Inherited and Acquired Kidney Diseases, Research Clinical Institute at Russian National Medical University, Moscow, Russia), Printza Nikoleta (Pediatric Nephrology Unit, 1st Pediatric Department, Aristotle University, Thessaloniki Greece), Prytula Agnieszka (Ghent University Hospital, Department of Pediatric Nephrology), Radermacher Luc (Service de néphrologie, CHR de Liège, Belgium), Quiroga Borja (Hospital Universitario de La Princesa, Madrid, Spain), Raluca Ene Lavinia (Centrul de diagnostic si tratament Dr Victor Babes, Bucuresti, Romania), Rapondzhieva Anushka Andreeva (Acibadem City Clinic Tokuda Hospital Sofia, Bulgaria), Roumeliotis Athanasios (Division of Nephrology and Hypertension, 1st Department of Internal Medicine, AHEPA Hospital, School of Medicine, Aristotle University of Thessaloniki), Roussinov Dimitar Lubomirov (Specialized Hospital for Active Treatment of Children's Diseases "Prof. Ivan Mitev ", Medical University, Sofia), Saltirov Iliya (Department of Urology and Nephrology, Military Medical Academy, Sofia, Bulgaria), Samadi Katayoon (Mashhad University of Medical Sciences Mashhad Iran), Schreuder Michiel F. (Radboudumc Amalia Children's Hospital, Nijmegen, The Netherlands), Sevinc Mustafa (Sisli Hamidiye Etfal Training and Education Hospital, Istanbul, Turkey), Shabaka Amir (Nephrology Department, Hospital Universitario Fundación, Alcorcón, Madrid, Spain), Sharma Jyoti (Pediatric Nephrology Service, Renal Unit, King Edward Memorial Hospital, Pune 411040, India), Sikora Przemyslaw (Department of Pediatric Nephrology, Medical University of Lublin, Poland), Simão Nunes Paula Alexandra (Hospital S.Francisco Xavier - Centro hospitalar de Lisboa Ocidental and Nova Medical School), Slotki Itzchak (Afek Medical Center Jerusalem, Israel), Soares dos Santos Augusto Cesar Junior (Hospital das Clinicas, Universidade Federal de Minas Gerais, Brazil, HC-UFMG/Ebserh), Soloukides Andreas (Transplantation Center and Nephrology Clinic, Nicosia General Hospital, Cyprus), Smith James R (Department of Renal Medicine, Aberdeen Royal Infirmary), Stamatelou Kyriaki (“Mesogeios” Nephrology Centers, Haidari, Greece), Stavroulopoulos Aristeidis (IASIO Hospital - General Clinic of Kallithea, Athens, Greece), Stepanova Natalia (Institute of Nephrology National Academy of Medical Science of Ukraine), Stoves John Bradford (Teaching Hospitals NHS Foundation Trust), Stylianou Kostas (Heraklion University Hospital Crete Greece), Tasic Velibor (Dept. of Pediatric Nephrology, University Children’s Hospital, 1000 Skopje, Macedonia), Tayfur Aslı Çelebi (University of Health Sciences, Ankara Keçiören Training and Research Hospital, Turkey), Teixeira Costa Fernando (Nephrology department, Hospital Garcia de Orta, Almada, Portugal), Teixeira França Ana Manuela Fernandes (Centro Materno-Infantil do Norte - Centro Hospitalar Universitário do Porto, Porto, Portugal), Tovbin David (Department of Nephrology, Emek medical Center, Afula, Israel), Van De Perre Els (Kidney Stone Prevention Clinic, Universitair Ziekenhuis Brussel, Vrije Universiteit Brussel, Brussels, Belgium), Varda Nataša Marčun (Unit for Paediatric Nephrology and Hypertension, Department of Paediatrics, University Medical Centre Maribor), Von Vigier Rodo O. (Pediatric Clinic, Wildermeth Children’s Hospital, Biel-Bienne, Switzerland), Wasilewska Anna (Pediatric Nephrology Department, Medical University of Białystok, Poland), Zanen Adriaan (Deventer Ziekenhuis, Deventer, The Netherlands Zanetta Gilbert, Néphrologie chu, Dijon 21034, France), Zaniew Marcin (Department of Pediatrics University of Zielona Gora, Zielona Gora, Poland), Zvenigorodska Ganna (Department of pediatrics №2, National Pirogov Memorial Medical University, Vinnytsya, Ukraine)
